# Irrigation modulates the effect of increasing temperatures under climate change on cotton production of drip irrigation under plastic film mulching in southern Xinjiang

**DOI:** 10.3389/fpls.2022.1069190

**Published:** 2022-12-12

**Authors:** Hongbo Wang, Zi Yin, Lei Zhang, Fengnian Zhao, Weixiong Huang, Xingpeng Wang, Yang Gao

**Affiliations:** ^1^ College of Water Hydraulic and Architectural Engineering, Tarim University, Alaer, China; ^2^ Laboratory of Modern Agricultural Engineering, Tarim University, Alaer, China; ^3^ Hubei Key Laboratory of Yangtze Catchment Environmental Aquatic Science, School of Environmental Studies, China University of Geosciences, Wuhan, Hubei, China; ^4^ Key Laboratory of Northwest Oasis Water-Saving Agriculture, Ministry of Agriculture and Rural Affairs, Shihezi, China; ^5^ Institute of Farmland Irrigation, Chinese Academy of Agricultural Sciences, Xinxiang, Henan, China

**Keywords:** climate change, AquaCrop model, irrigation quota, warming, predicting yield

## Abstract

**Introduction:**

Warming and drought brought about by climate change seriously harm sustainable agricultural production in southern Xinjiang. It is still unclear how irrigation can improve the ability of crops to cope with climate change.

**Methods:**

Therefore, in this study, we calibrated and validated the AquaCrop model using data collected in cotton production from 2017 to 2018. The model effectively simulated the growth, biomass, and yield of cotton plants at the experimental site under different warming and irrigation conditions. The meteorological data collected from 1987 to 2016 were used in a simulation to predict cotton production under 3 temperature scenarios (temperature increased by 0°C, 1°C, and 2°C) and 6 levels of irrigation (198, 264, 330, 396, 495, and 594 mm) to explain the modulating effect of plastic film mulching-coupled drip irrigation on cotton production in terms of increasing temperatures under climate change in southern Xinjiang.

**Results and discussion:**

Model prediction showed that an increase in temperature reduced cotton yield under a low irrigation level, while an increase in irrigation mitigated the impact of climate change on cotton yield. An increase of 1°C did not significantly reduce cotton yield at 198–330 mm of irrigation. Under a 2°C increase, 396–594 mm of irrigation was required to ensure plant growth and yield formation. Both aboveground biomass and yield increased with the rise in the irrigation level at the same temperature. High water use efficiency was achieved at 495 mm of irrigation without significant yield loss. Therefore, in the low-temperature scenario, it can be preferentially considered to achieve sustainable water use through water management, while in the high-temperature scenario innovative agricultural measures are required to avoid yield loss. Optimizing irrigation strategies can reduce warming-induced damage to crops under climate change.

## 1 Introduction

Climate changes, characterized by temperature rise, the uncertain amount and patterns of precipitation, and elevated atmospheric CO_2_ concentration (Piao et al., 2010), are widely concerned issues in global agricultural development (Nie et al., 2022). Studies have shown that world agricultural production growth is expected to decrease at an annual rate of 1.5% by the year 2030 and then a further reduction of 0.9% by 2050, compared with 2.3% growth per year since 1961 (Dubey and Sharma, 2018). The impact of climate change on crop yields was mainly reflected in temperature increase, with an average yield loss of 2.58% per°C at the national level in China. Subregional yield changes ranged from -12.70% to -2.57% per°C, with crop yields being more vulnerable in Northeast China and Northwest China than in other subregions (Liu et al., 2020). Probably the most common strategy for addressing current and future climate change-related challenges and adapting proper agricultural practices is to focus primarily on improving agricultural water productivity by changing the planting date, crop variety, and irrigation water-saving approach (Davarpanah and Ahmadi, 2021). However, the drivers (greenhouse gases) and impacts of climate change (rainfall variability and increasing temperature) are projected to evolve with time, thereby determining their effect on plants’ phenology and yield production is likely to become more complex in the future (Poulter et al., 2013). Hence, there is a need to enhance the understanding of climate impacts on agricultural systems to better manage crops and mitigate the effects of future climate change (Anwar et al., 2020).

Crop models coupled with long-term weather data provide an opportunity for evaluating yield variability by simulating numerous potential scenarios (Masasi et al., 2020). Crop models can be used to understand the effects of climate change, such as the choice of crop’s optimum planting date (Kazeem and Rasaq, 2015), the creation of an irrigation schedule ensuring increased water productivity (Linker et al., 2016; Tsakmakis et al., 2018), and the assessment of climate change impacts on crop yield (Voloudakis et al., 2015). Based on the APSIM model, Chen et al. (2019) concluded that future climate scenarios (RCP 4.5 and RCP 8.5) might lead to cotton yield increase because the carbon fertilization effect mitigated low-temperature stress, thereby slightly increasing cotton yield. Chen et al. (2011) used the COSIM cotton model to simulate cotton production under SRES A2 and B2 emission scenarios and concluded that planting cotton in the Shiyang River basin would be expanded in the future, as there is a certain potential for cotton production. Based on the DSSAT model, Adhikari et al. (2016) found that under climate change, the cotton yield would decrease by 2.0%~14.9% when disregarding the increase of CO_2_ concentration; however, while considering the increase in CO_2_ concentration, the cotton yield would increase by 30%~53%. Studies have shown that the intensity and direction of climate change impacts on crop production are complex and uncertain and may result in net positive or negative outcomes (Irmak et al., 2022). Generally, climate change is expected to put pressure on crop production and has already caused yield losses (Lobell et al., 2011; Asseng et al., 2014). Increased frequency of co-occurring high temperatures and shortage of water (Alizadeh et al., 2020) suppresses crop yields by causing heat and water stress in crops (Lesk et al., 2016). The climate impacts are crop and site-specific, and cannot be generalized for different regions/crops; thus, reiterating the need to conduct crop and site-specific impact assessments (Rashid et al., 2019).

Located in the hinterland of Eurasia, southern Xinjiang has a typical continental arid climate and features sufficient sunlight, abundant heat, scarce precipitation, and dry air (Hu et al., 2019). Southern Xinjiang’s unique climate environment is conducive to the growth of cotton, high quality, and high yield (Wang et al., 2020). However, the extreme warm events (2017–2035), poor temperature index, warm days, and extreme maximum temperature will increase in the Tarim River basin in the future. In addition, the drought in the central part of the basin may be more severe, while the mountainous areas around the Tarim Basin will tend to become wet (Chen et al., 2017), which may bring new challenges to the sustainability of regional crops (Li et al., 2019). In face of increasing water shortage, climate change, and climate change uncertainty, increasing agricultural water efficiency and productivity is needed to reduce negative environmental impacts (Vanuytrecht et al., 2014; Alvar-Beltrán et al., 2021). Therefore, to improve irrigation efficiency, irrigation techniques and irrigation strategies should be used as a means to improve overall irrigation management, to make rational use of the existing water resources (Li et al., 2019).

This paper takes the mulched cotton by drip irrigation in the oasis as the study object, mainly studying the following contents: (1) the model parameters were localized in this study, and the applicability of the AquaCrop model in the simulation of cotton growth and yield was verified. (2) By changing input parameters, the effects of warming and irrigation on cotton growth, biomass accumulation, and yield formation under climate change were simulated, and the impact of the environment on cotton production was determined. (3) This study identifies the optimal irrigation scheme for cotton planting systems under climate change to make rational use of existing water resources and provide a theoretical basis for sustainable cotton production under future climate change.

## 2. Materials and methods

### 2.1 Experimental site

The experiment was conducted at the Soil and Water Conservation Monitoring Station (81°17′56″E, 40°32′36″N, altitude 1100 m.a.s.l) of the 1^st^ Division of Xinjiang Production and Construction Corps, China. The region has a typical inland extremely arid climate, with an annual average precipitation of 50 mm, annual evaporation of 2218 mm, and an annual average temperature of 11.3°C. The soil is sandy loam, which is uniform in the depth of 0~100 cm, with the bulk density of 1.58 Mg m^-3^, and the field capacity of 0.24 g g^-1^. The electrical conductivity is about 2 dS cm^-1^. The groundwater table is on average 3 m below the soil surface.

### 2.2 Experimental design

The randomized complete block design experiment with three levels of irrigation was carried out in the 2017 and 2018 seasons. From the beginning of the cotton budding period, irrigation would be applied when the difference between crop evapotranspiration (*ET_C_*, mm) and precipitation (*P*, mm) reaches 30 mm (Fan et al., 2019). At present, the irrigation treatment of cotton under film-mulched drip irrigation in southern Xinjiang is around 30 mm, which is slightly different among regions. Therefore, three irrigation levels were designed as: T1: 30 × 0.8 = 24 mm, T2: 30 × 1.0 = 30 mm, T3: 30 × 1.2 = 36 mm, respectively. Irrigation scheduling for the three treatments in 2017 and 2018 is shown in **Table 1**. Each treatment had three replicas, and each plot area was 154 m^2^ in size (length of 22 m and width of 7 m, respectively).

**Table 1:** Irrigation scheduling of cotton in 2017 and 2018.

Date		Irrigation treatment (mm)		
2017	2018	T1	T2	T3
6/7	6/16	24	30	36
6/17	6/26	24	30	36
6/23	7/6	24	30	36
7/3	7/13	24	30	36
7/10	7/19	24	30	36
7/14	7/26	24	30	36
7/25	8/3	24	30	36
7/31	8/8	24	30	36
8/6	8/14	24	30	36
8/13	8/20	24	30	36
8/20	8/26	24	30	36
Total amount (mm)		264	330	396

**Figure 1:**
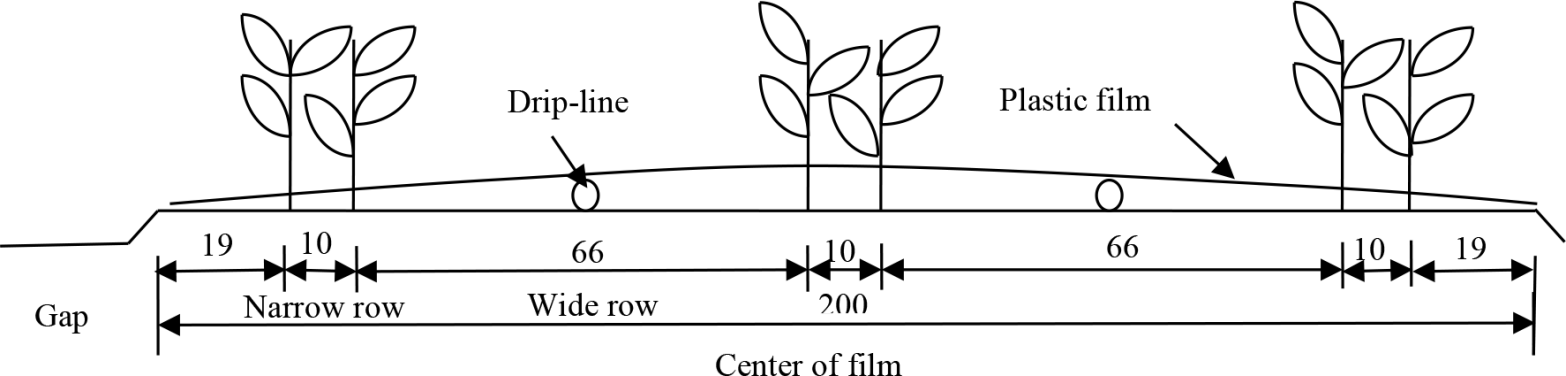
Schematic diagram of cotton planting pattern (unit:cm).

The cotton (*Gossypium hirsutum* L.) variety was “Xinluzhong 46” with a planting density of 1.6×10^3^ plants hm^-2^. In 2017, the sowing date of cotton under film mulched drip irrigation was April 3, and the harvest was completed on October 1. In 2018, the sowing date was April 15, and the harvest was completed on October 12. The planting pattern of cotton under film-mulched drip irrigation is shown in **Figure 1**. The drip-line diameter was 16 mm, and the emitter spacing was 20 cm. The flow rate was 3.0 L h^-1^ with a pressure of 0.1 MPa. Fertilization, pesticide spraying, and other agronomic measures were carried out according to local practices.

### 2.3 Modeling methodology

#### 2.3.1 Essence of AquaCrop

AquaCrop consists of atmosphere modules, soil modules, crop modules, and management modules. The AquaCrop model can simulate biomass and yield production based on the amount of water transpired from the green canopy cover under the governing environmental conditions (Raes et al., 2009).

The final seed cotton yield (*Y*) is derived as the product of Biomass (*B*) on the harvesting day, and the harvest index (*HI*), i.e., the percentage ratio of seed cotton yield to aboveground dry biomass, as:


(1) Y=B×HI

Biomass is estimated throughout the growing season as the product of water productivity (WP*) and the ratio of daily transpiration (*T_r_*) and reference evapotranspiration (*ET_0_*):


(2) $$B=WP^{*}\times \sum^{}\left(\frac{T_{r}}{ET_{0}}\right)$$

The model calculates the daily transpiration *T_r_* according to an empirical parameterization, as:


(3) $$T_{r}=K_{S}\times CC^{*}\times K_{CT_{r,x}}\times ET_{0}$$

where, *K_S_* represents the soil water crop coefficient integrating water logging, stomatal closure and early senescence effects; CC* is the canopy cover adjusted for micro-advective effects; *K_CTr,x_* is the crop coefficient for maximum crop transpiration (determined by the characteristics that distinguish a crop with a complete canopy cover from the reference grass.

#### 2.3.2 Meteorological parameters

**Figure 2:**
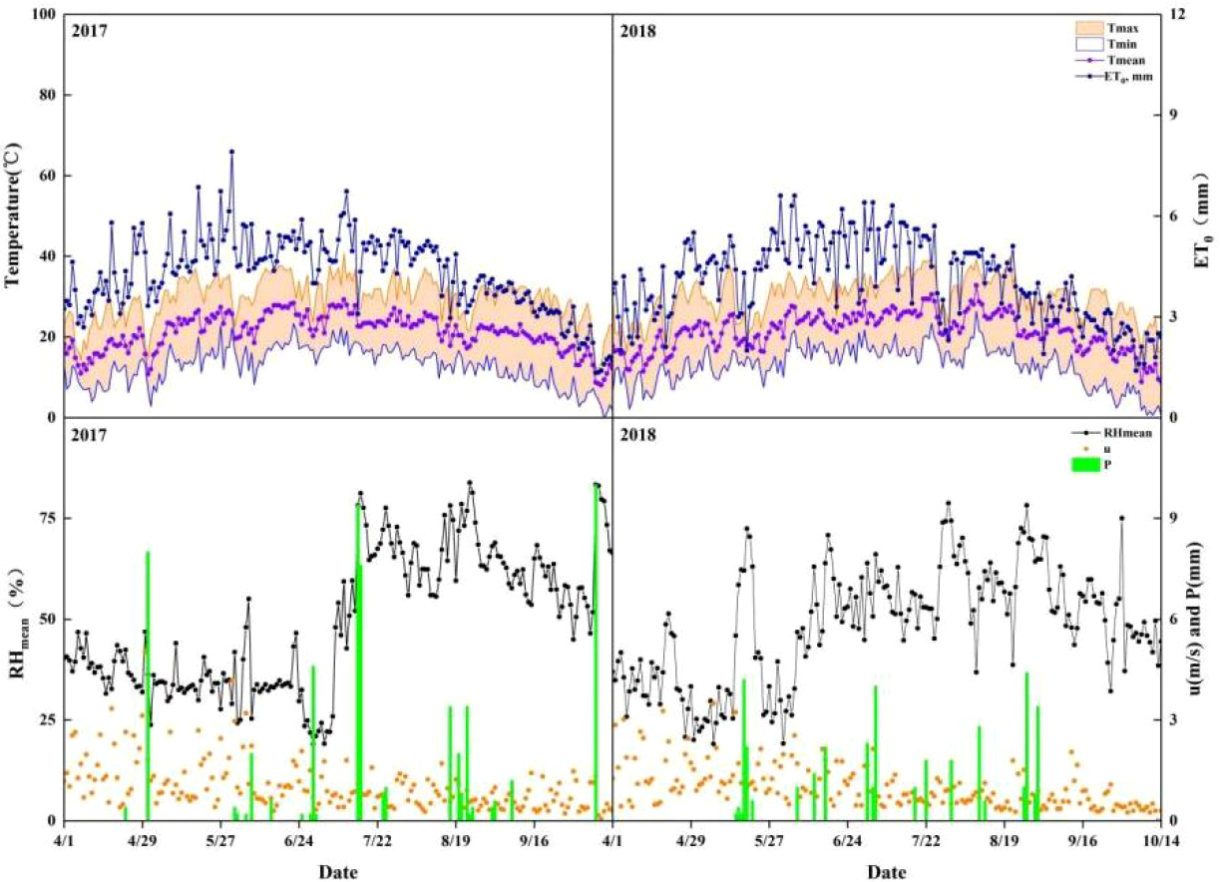
Meteorological data in 2017and 2018. *ET_0_*: Reference crop evapotranspiration; *RH_mean_*: Relative humidity; *u*: Wind speed; *P*: Precipitation.

Meteorological data were continuously measured during the experimental period by a standard automatic weather station (Hobo, Onset Computer Corp., USA) located near the experimental field. The data were taken every 5 s, and 15 min averages were recorded by a data logger. The *ET_0_* was calculated using the FAO Penman-Monteith method. Meteorological data in the 2017–2018 seasons are shown in **Figure 2**.

#### 2.3.3 Crop parameters

The AquaCrop crop growth simulation model was used to assess the response of cotton to climate change. According to the calibration procedure of Vanuytrecht et al. (2014), canopy cover, aboveground biomass, and yield were calibrated in order. For cotton under film-mulched drip irrigation, the model was calibrated using the experimental data in 2018 and validated using the data in 2017. The crop parameters are shown in **Table 2**.

**Table 2:** Crop parameters of AquaCrop model.

Description and unit	Value	
	Default AquaCrop file	Calibrated value
Decline in crop coefficient after reaching, %	98	90
Canopy decline coefficient at senescence, %/d^-1^	2.9	5.2
Maximum crop coefficient	1.10	1.15
Maximum effective rooting depth	2	0.8
Water productivity normalized for *ET_0_* and CO_2_, g m^-2^	15	18
Reference harvest index, %	35	34
Leaf growth threshold p-upper	0.20	0.35
Leaf growth threshold p-lower	0.70	0.65
Stomatal conductance threshold p-upper	0.65	0.35
Soil water depletion threshold for senescence acceleration	0.75	0.60
Base temperature *T*_base_, °C	12	15
Upper temperature *T*_upper_, °C	35	35

#### 2.3.4 Soil data

The physical soil characteristics were directly measured in the field and input into AquaCrop (**Table 3**). Additionally, there is no impermeable layer near the soil surface, and the groundwater table depth is 3 m. The parameters were input into the AquaCrop model, and a soil data file was generated.

**Table 3:** Soil physical properties.

Soil depth/cm	Bulk density/(Mg m^-3^)	Field capacity/(g g^-1^)	Saturated water content/(g g^-1^)	Permanent withering coefficient/(g g^-1^)	Cosmid/%	Powder/%	Grit/%
0-20	1.60	0.21	0.24	0.10	2.43	41.49	56.08
20-40	1.55	0.24	0.30	0.10	2.56	41.40	56.05
40-60	1.58	0.25	0.33	0.12	2.88	42.82	54.29
60-80	1.59	0.25	0.32	0.13	2.60	41.40	56.00

### 2.4 Scenario simulation

According to the Fifth Assessment Report of the Intergovernmental Panel on Climate Change (IPCC) and related research reports, in Northwest China, under extreme conditions, the future average temperature change will be around 1.5–2°C, and the future average precipitation change will range between 10% and 20% by the end of the 21st century (2081–2100) (IPCC, 2013). Therefore, in this study, we calibrated and validated a model based on the meteorological data collected from 1987 to 2016 and used the model in a simulation with 6 irrigation levels and 3 warming treatments, for a total of 18 scenarios (**Table 4**). The irrigation levels were TS1 (30×0.6 = 18 mm), TS2 (30×0.8 = 24 mm), TS3 (30×1.0 = 30 mm), TS4 (30×1.2 = 36 mm), TS5 (30×1.5 = 45 mm), and TS6 (30×1.8 = 54 mm). The warming treatments were W1 (+ 0°C), W2 (+ 1°C), and W3 (+ 2°C).

**Table 4:** Simulation scenarios.

Simulation scenarios	Irrigation treatment TS/mm	WarmingW	Simulation scenarios	Irrigation treatment TS/mm	warmingW
P_1_	18	+0°C	P_10_	36	+0°C
P_2_		+1°C	P_11_		+1°C
P_3_		+2°C	P_12_		+2°C
P_4_	24	+0°C	P_13_	45	+0°C
P_5_		+1°C	P_14_		+1°C
P_6_		+2°C	P_15_		+2°C
P_7_	30	+0°C	P_16_	54	+0°C
P_8_		+1°C	P_17_		+1°C
P_9_		+2°C	P_18_		+2°C

### 2.5 Statistical analysis

The outputs of the AquaCrop model were assessed with the root mean square error (*RMSE*), normalized root mean square error (*NRMSE*), Synergy index (*d*), and relative error (*RE*), which were calculated from Eq (4) to Eq (7) (Young et al., 2017).


(4) $$RMSE={\left[\sum_{i=1}^{n}\frac{{\left(P_{i}-Q_{i}\right)}^{2}}{n}\right]}^{0.5}$$

(5) $$NRMSE={\left[\sum_{i=1}^{n}\frac{{\left(P_{i}-Q_{i}\right)}^{2}}{n}\right]}^{0.5}\times \frac{100}{\bar{O}}$$

(6) $$d=1-\left[\frac{\sum_{i=1}^{n}{\left(P_{i}-Q_{i}\right)}^{2}}{\sum_{i=1}^{n}{\left(\left|P_{i}-\bar{O}\right|+\left|O_{i}-\bar{O}\right|\right)}^{2}}\right]$$

(7) $$RE=\frac{P_{i}-Q_{i}}{Q_{i}}\times 100$$

where, *P_i_* is the predicted value, *O_i_* is the measured value, *Ō* is the average of the measured values, and *n* is the number of samples.

## 3 Results

### 3.1 Model calibration and validation

Canopy cover, aboveground biomass, soil moisture, crop yield, and *ET* were calibrated using the experimental data in 2018. The statistical indicators for calibration errors are shown in **Table 5**. Model accuracy for canopy cover, aboveground biomass, and soil moisture in 2018 was relatively high, *NRMSE* and *RE* for canopy cover, aboveground biomass, and soil moisture were both<15% for all treatments, and that *d* and *R* ^2^ were both close to 1. For the predicted crop yield and *ET*, *NRMSE* and *RE* were both<10%, with *d* and *R* ^2^ both relatively low. Comprehensive analysis showed that the predicted and measured values for 2018 were in very good agreement.

**Table 5:** Calibration and validation of AquaCrop model.

Indicator	Treatment	*RMSE*		*NRMSE* (%)		*d*		*R* ^2^		*RE* (%)	
		2017a	2018a	2017a	2018a	2017a	2018a	2017a	2018a	2017a	2018a
Canopy cover (%)	T_1_	5.90	2.95	10.00	4.86	0.98	1.00	0.98	0.99	13.66	5.61
	T_2_	2.33	3.71	3.71	6.07	1.00	0.99	1.00	0.98	4.14	4.82
	T_3_	5.85	2.80	9.50	4.48	0.99	1.00	0.98	0.99	12.66	3.68
Biomass	T_1_	618.54	73.44	10.36	1.23	0.99	1.00	0.99	1.00	4.17	-0.41
	T_2_	903.82	374.62	14.98	6.24	0.98	1.00	0.99	0.99	7.97	3.68
	T_3_	1071.81	802.99	17.36	12.57	0.98	0.98	0.96	0.94	14.27	3.34
Soil moisture (vol%)	T_1_	1.09	0.90	5.63	4.93	0.91	0.91	0.85	0.72	-1.46	-0.28
	T_2_	0.98	1.15	4.63	5.82	0.95	0.89	0.88	0.74	1.14	1.26
	T_3_	1.37	1.56	5.89	7.28	0.90	0.87	0.72	0.66	1.66	3.29
Yield (kg hm^-2^)	T_1_	560.12	415.56	9.42	7.82	0.38	0.35	0.81	0.82	-8.60	7.39
	T_2_	481.99	539.85	7.27	9.33	0.30	0.25	0.81	0.82	-7.00	9.18
	T_3_	558.72	355.01	7.90	5.85	0.42	0.30	0.81	0.82	-6.79	5.66
ET(mm)	T_1_	13.34	34.82	3.14	9.23	0.12	0.28	0.87	0.87	-0.26	9.02
	T_2_	11.99	29.98	2.58	7.09	0.41	0.15	0.87	0.87	1.24	6.87
	T_3_	27.33	17.35	5.20	3.59	0.41	0.44	0.87	0.87	-4.51	-2.85

The AquaCrop model was validated using the experimental data in 2017, and the statistical indicators for each treatment are shown in **Table 5**. For canopy cover, *RMSE* for different treatments was in the range of 2.33%~5.90%, and *NRMSE* was in the range of 3.71%~10.00%; *d* and *R^2^* were in the range of 0.98~1.00. For aboveground biomass, *RMSE* for the different treatments was in the range of 628.54~1071.81 kg hm^-2^, *NRMSE* was in the range of 10.36%~17.36%, *d* was in the range of 0.98~0.99, and *R^2^* was in the range of 0.96~0.99. The comparative analysis of simulated and measured soil moisture contents shows acceptable fitness both for different treatments with the *RMSE* of 0.98%~1.37%, *NRMSE* of 4.63%~5.89%, *d* of 0.90~0.95 and *R^2^* of 0.72~0.88, respectively. *NRMSE* was<20% for both measured and predicted values in each treatment, and *d* and *R* ^2^ were both close to 1. These values indicate that the AquaCrop model well represents the dynamic changes in canopy cover, aboveground biomass, and soil moisture.

By correlating the observed and simulated actual evapotranspiration (*ET*) and cotton yield, *RE* for the different treatments was -4.51%~1.24% and -8.60%~-6.79%. Comprehensive analysis showed that the AquaCrop model underestimated *ET* and cotton yield, meanwhile overestimated canopy cover, aboveground biomass, and soil moisture. However, it testifies to the calibration and validation accuracy of the AquaCrop model scenarios analyses.

### 3.2 Atmospheric temperature change and the number of affected days during the cotton growth period under climate change

**Figure 3:**
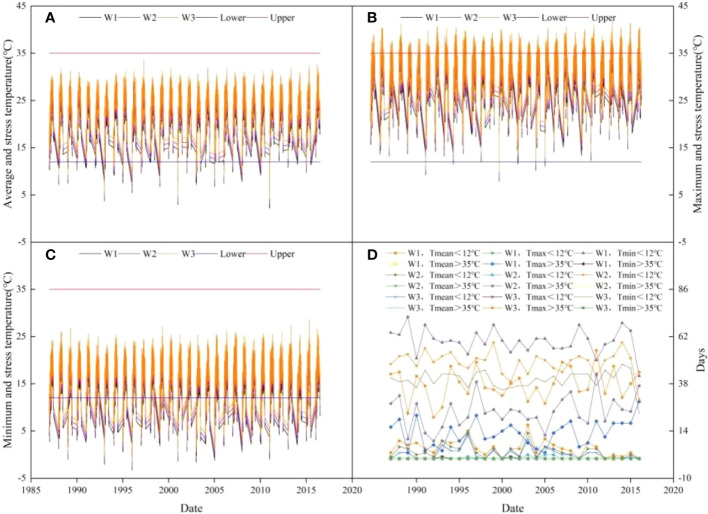
Atmospheric temperature change and the number of days that are affected by temperature stress during the cotton growth period under climate change from 1987 to 2016. **(A)** Average and stress temperature. **(B)** Maximum and stress temperature. **(C)** Minimum and stress temperature. **(D)** Stress days.

Low temperatures (<12°C) occurring in the early growth stage stunt cotton plant growth. High temperatures (>35°C) occurring in the mid-growth stage adversely affect pollen viability, cotton boll size, the number of seeds, and shedding of flower buds and young cotton bolls, thereby reducing water use efficiency and cotton yield (Pettigrew, 2008; Li et al., 2020). The mean temperature (*T_mean_*), maximum temperature (*T_max_*), minimum temperature (*T_min_*), and the number of temperature-affected days during the growing period of cotton plants collected from 1987 to 2016 are shown in **Figures 3A–D**. In W1, which had no warming treatment, 5 and 60 days had an average *T_mean_* and *T_min_* lower than 12°C, respectively; none of the days had a *T_mean_* or *T_min_* higher than 35°C, and *T_max_* was higher than 35°C and lower than 12°C for 13 and 0 d, respectively. The number of days for cotton plants to be exposed to low temperatures (<12°C) decreased with the increase in *T_mean_* and *T_min_*. Compared with W1 (no warming), W2 (1°C increase) and W3 (2°C increase) increased *T_mean_* by 34.15% and 54.88% and *T_min_* by 18.13% and 32.96%, respectively. The number of days that cotton plants were exposed to high temperatures (>35°C) was not affected by the increase of *T_mean_* and *T_min_*. With the increase in *T_max_*, the number of days that cotton plants were exposed to high temperatures increased. W2 and W3 increased the number of days in which cotton plants were exposed to high temperatures by 82.65% and 188.27%, respectively, compared with W1. The exposure time of cotton plants to low temperatures was not affected by the increase in *T_max_*.

### 3.3 Effect of warming and irrigation on the biomass, yield, water consumption, and water use efficiency of cotton plants under climate change

**Figure 4:**
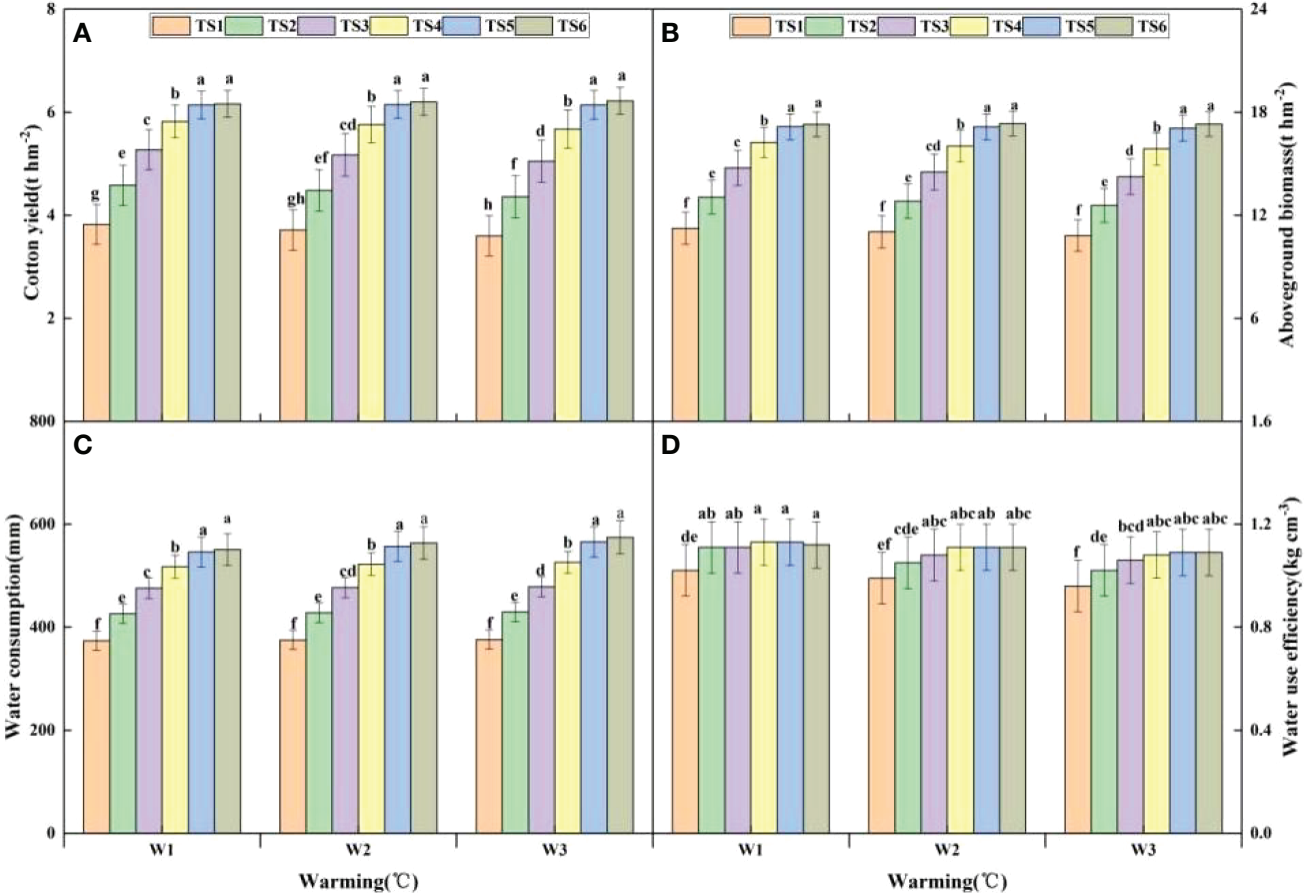
The effects of irrigation and warming on cotton biomass, yield, water consumption, and water use efficiency under climate change. TS1: 18 mm; TS2: 24 mm; TS3: 30 mm; TS4: 36 mm; TS5: 45 mm; TS6: 54 mm. **(A)** Cotton yield. **(B)** Aboveground biomass. **(C)** Water consumption. **(D)** Water use efficiency. Lowercase letters a–f indicate the significant difference among treatments at 0.05 level.

The effects of warming and irrigation on cotton yield under climate change are shown in **Figure 4A**. Cotton yield increased with an increase in irrigation levels at different temperatures. The cotton yield under irrigation levels TS2–TS6 was 20.48%, 39.03%, 55.00%, 65.57%, and 66.93% higher, respectively than that under TS1. In TS5 and TS6, the cotton yield was significantly higher than that in TS1–TS4, and the difference between TS5 and TS6 was not significant. Cotton yield decreased with the increase in temperature under irrigation levels TS1–TS4, and W2 and W3 reduced cotton yield by 2.40% and 4.92%, respectively, compared with W1. In contrast, the yield in TS5 and TS6 increased with the temperature rise, and W2 and W3 increased the cotton yield by 0.52% and 0.51%, respectively, compared with W1. In the TS1–TS3 irrigation levels, W3 significantly reduced cotton yield compared with W1, while it was not significantly affected by warming in TS4–TS6.

Aboveground biomass is an important indicator of plant adaptation to environmental factors and provides a basis for determining the optimal allocation of photosynthetic products between vegetative and reproductive growth. **Figure 4B** indicates that the changing trend of cotton biomass is consistent with that of the yield at the same temperature. Compared with TS1, TS2–TS6 increased biomasses by 19.19%, 36.86%, 52.37%, 62.92%, and 64.49%, respectively. Cotton biomass significantly increased in TS5–TS6 compared with TS1–TS4. The biomass showed an overall decreasing trend with the increase in temperature, except for a significant difference with the temperature change in irrigation level TS3. Overall, a high irrigation level favored the formation of cotton biomass and yield, and elevated temperatures only adversely affected the formation of biomass and yield at a low irrigation level.

The effects of warming and irrigation on the water consumption of cotton plants under climate change are shown in **Figure 4C**. Water consumption increased with an increase in irrigation level at all temperatures. TS5 and TS6 significantly increased cotton yield compared with TS1–TS4, and there was no significant difference in yield between TS5 and TS6. The water consumption of cotton plants increased with an increase in temperature at different irrigation levels. W3 significantly increased water consumption compared with W1 in irrigation levels TS5–TS6, while water consumption in TS1–TS3 quotas was not significantly affected by temperature. **Figure 4D** indicates that the change trends in water use efficiency and water consumption at different temperatures were the same. At W1 and W3 temperatures, TS2–TS6 significantly increased water use efficiency compared with TS1. However, the water use efficiency under different irrigation levels was reduced with an increase in temperature. At the TS1 irrigation level, W1 significantly increased water use efficiency compared with W3.

## 4. Discussion

The impact of climate change on crop production can be revealed using experimental, modeling, and analytical approaches (Ahmadi et al., 2015; Lenka et al., 2019; Rashid et al., 2019; Lenka et al., 2021). Considering the complexity of farmland ecosystems and the limitations of field experiments, using crop models to simulate the growth process and yield of crops is an important way to cope with the impact of climate change and human activities on agricultural production and to achieve water saving and yield increase (Akumaga et al., 2017; Zhao et al., 2018);. In our study, the AquaCrop model was used to evaluate the impact of irrigation and warming on cotton yield in the oasis area under climate change, and the accuracy of the calibration and validation of the AquaCrop model was validated for scenario analysis. The results show that *NRMSE* for Canopy cover, aboveground biomass, and soil moisture was<20% for all treatments and that d and R^2^ were both close to 1. *REs* for *ET* and seed cotton yield predictions were -4.51~1.24% and -8.60~-6.79%, respectively. Voloudakis et al. (2015) used the AquaCrop model to simulate the response of the yield of Greek cotton to different climate scenarios, which provided a basis for formulating future irrigation plans in the area. In addition, based on 8 climate factors under the IPCC AIB emission scenario, the AquaCrop model was used to analyze the relationship between cotton yield and climate change, and the prediction showed an upward trend in cotton yield (Voloudakis et al., 2018). Based on the AquaCrop model, Li et al. (2021) concluded that for every 1°C increase in the mean temperature in Xinjiang, cotton yield decreased by 1.64%, and for every 1% increase in precipitation and 1 ppm increase in CO_2_ concentration, cotton yield increased by 0.09% and 0.05%, respectively. These studies demonstrate that the AquaCrop model is applicable for simulating climate change, and the results may vary with different regions, climates, soil textures, and irrigation water quality.

The main biophysical processes of crop production, such as soil evaporation, plant evapotranspiration, nutrient cycling, and plant growth (Durodola and Mourad, 2020) largely depend on climatic conditions and fluctuations (Kheir et al., 2019). Based on the analysis of meteorological data collected from 1987 to 2016, during the cotton growth period, the number of days in which the average *T_mean_* and *T_min_* were lower than 12°C (low-temperature stress) was 5 and 60 d, respectively. A *T_mean_* and *T_min_* higher than 35°C (high-temperature stress) were not observed. *T_max_* was higher than 35°C on 13 d, but no days had a *T_max_* lower than 12°C. There are large temperature differences between day and night in southern Xinjiang, and for about one-third of the growth period, cotton plants are subjected to low-temperature stress at night. Drip irrigation underneath plastic mulch film not only increased soil temperature and preserved moisture but also contributed to the cumulative air temperature required for the growth of cotton plants (Zumilaiti et al., 2018), which mitigated the impact of low temperature on cotton growth and yield. Meanwhile, climate change leads to warm and dry weather, which reduces the number of days in which cotton plants are exposed to low temperatures (<12°C) but increases the number of days they are subjected to high temperatures (>35°C).

Climate change has dramatically increased the demand for water in agricultural production in southern Xinjiang. In this situation, an adjustment in irrigation strategy is required to cope with the impact of climate change and to reduce the water demand in agricultural irrigation. The cotton yield under irrigation levels TS1–TS4 showed a decreasing trend with an increase in temperature, indicating that warming might directly affect cotton yield by impairing morphological development and plant growth (Anwar et al., 2020).Cotton yield in irrigation levels TS5–TS6 increased with the temperature rise, and the difference between them was not significant, indicating that irrigation was effective for increasing yield and mitigating high-temperature stress, which could be used to directly relieve water stress on crops and indirectly reduce heat stress to reduce the dependence of crop yield on climatic conditions and even reverse the response in some cases (Luan et al., 2021). Warming might directly affect crop evapotranspiration and the need for crops for irrigation water (Nie et al., 2022). In our study, evapotranspiration increased with the rise in temperature because, at high temperatures, air can hold more water, increasing the potential for evapotranspiration (Muluneh, 2020). Therefore, under low-temperature conditions, water should be saved for sustainable water use through improving water management. Under high-temperature conditions, innovative agricultural measures are required to reduce yield loss, and an optimized irrigation strategy is needed to mitigate warming-induced damage to crops under climate change.

Determining the effect of weather conditions on crop growth based on historical data is of great significance for guiding future agricultural production. In our study, data collected from 1987 to 2016 were used to simulate cotton production under 18 weather scenarios. Cotton biomass and seed cotton yield increased with the increase in irrigation level, and there was no significant difference in cotton biomass and seed cotton yield between TS5 and TS6. Similarly, Tan et al. (2018) concluded that irrigation levels around 406–462 mm were appropriate for cotton production equipped with drip irrigation and plastic film mulching in southern Xinjiang, and variations in climate, soil texture, and irrigation water quality might lead to some discrepancies. Excessive or inadequate irrigation was not helpful, while an appropriate amount of irrigation was useful for improving cotton yield. This is because an appropriate amount of water is conducive to the accumulation of aboveground biomass, while excessive irrigation causes fertilizer leaching that leads to low efficiency in fertilizer absorption and utilization, reducing the vegetative and reproductive growth of plants and thereby reducing cotton yield (Cui et al., 2018). Both the water consumption and water use efficiency of cotton plants increased with an increase in irrigation level, which was consistent with the change in yield. This stems from the fact that cotton yield is decided by biomass and harvest index, and cotton biomass and water consumption are closely related (Paredes et al., 2015). In our study, TS1–TS3 irrigation levels mitigated the adverse effect of a 1°C increase on cotton production, while increased irrigation levels (TS4–TS6) were required for the growth and yield formation of cotton plants when the temperature increased by 2°C.

Increasing irrigation is a technical measure that is very effective in adapting to the changing climate as shown in a paper (Luan et al., 2021). However, in many regions, irrigation is unsustainable to expand or impossible to implement due to water scarcity (Rosa et al., 2020). This may mean that increasing irrigation leads the agricultural system down a cul-de-sac (Bird et al., 2015). Therefore, the adaptation of agricultural practices perse, such as shifting sowing time, changing cultivars, and land use options, should also be explored as regional strategies to minimize the overall impact of global warming on cotton production (Teixeira et al., 2013).

## 5 Conclusions

Calibration and validation of the AquaCrop model showed that the model could accurately simulate cotton canopy cover, aboveground biomass, and seed cotton yield, which suggests that the AquaCrop model can be suitably adapted for use in an oasis area. The model prediction indicated that the aboveground biomass and yield of cotton under the same warming dates increased with the increase in irrigation levels. At an irrigation level of 495 mm, higher irrigation water efficiency was obtained, and it is ensured that no significant reduction in cotton yield occurred. Warming will reduce cotton production under the low irrigation level while increasing the irrigation level can reduce the dependence of cotton yield on climate change and improve the temperature resistance of cotton. This study will supply useful knowledge about the impact of different irrigation schedules on crop growth under future climate, and help to optimize the selection of feasible irrigation schedules to balance the relationship between water scarcity and dependable crop yield.

## Data availability statement

The raw data supporting the conclusions of this article will be made available by the authors, without undue reservation.

## Author contributions

HW: investigation, data analysis, writing—original draft. ZY, LZ and FZ: methodology, software, data analysis. WH: conceptualization, writing—review and editing. XW: resources, funding acquisition, supervision, project administration. YG: idea, formal analysis, writing—review and editing. All authors contributed to the article and approved the submitted version.
